# Supplementary material for: Diversity of Arbuscular Mycorrhizal Fungi of the Rhizosphere of Lycium barbarum L. from Four Main Producing Areas in Northwest China and Their Effect on Plant Growth
Source: J Fungi (Basel). 2024 Apr 12;10(4):286. doi: 10.3390/jof10040286 (PMC11050802; doi:10.3390/jof10040286)
Supplement: Supplementary file 1 [file jof-10-00286-s001.zip › jof-2932075-supplementary.pdf]

Supplementary material

Table S1 Modified Hoagland's nutrient solution formulation

Distribution of the proportion of each element
$Ca(NO_3)_2 \cdot 4H_2O$ 945 mg/L，KNO_3 506 mg/L，NH_4NO_3 80 mg/L，KH_2PO_4 136 mg/L，$MgSO_4$ 493 mg/L，Iron salt solution 2.5 m1 (Pay attention to the concentration of mother liquor)，Trace element liquid 5 ml，pH=6.0.
Iron salt solutions include $FeSO_4 \cdot 7H_2O$ 2.78 g，EDTA-2Na 3.73 g，dH_2O 500 m1，pH=5.5.
Trace element liquid include KI 0.83 mg/L，H_3BO_3 6.2 mg/L，$MnSO_4$ 22.3 mg/L，$ZnSO_4$ 8.6 mg/L，Na_2MoO_4 0.25 mg/L，$CuSO_4$ 0.025 mg/L，$CoCl_2$ 0.025 mg/L。
Except for iron salts and trace elements, it is not recommended to prepare the master batch because it is often accompanied by flocculent precipitation.

Note: The nutrient supply should be diluted or reduced according to the formula for expansion or growth promotion benefit testing.

Table S2 Classification of AMF species for morphological identification 1

Species	Regions				IF	RA	IV
	ZN	GY	JH	DL			
Glomus aureum	+				0.82%	2.11%	1.46%
Glomus badium	+				1.22%	3.16%	2.19%
Glomus coronatum	+				0.82%	2.11%	1.46%
Glomus deserticola				+	0.82%	3.51%	2.16%
Glomus etunicatum		+		+	6.33%	14.49%	10.41%
Glomus globiferum				+	4.49%	19.30%	11.89%
Glomus intraradices		+			3.06%	15.00%	9.03%
Glomus melanosporum	+		+		2.04%	2.73%	2.39%
Glomus microcarpum	+				1.63%	4.21%	2.92%
Glomus minutum	+	+			2.24%	3.79%	3.02%
Glomus monosporum		+			1.02%	5.00%	3.01%
Glomus mosseae	+				2.04%	5.26%	3.65%
Glomus pansihalos	+		+	+	2.86%	2.92%	2.89%
Glomus pustulatum			+		1.22%	3.41%	2.32%
Glomus reticulatum	+		+		3.27%	4.37%	3.82%
Rhizophagus aggregatus	+				1.22%	3.16%	2.19%
Septoglomus constrictum	+				0.82%	2.11%	1.46%
Scutellospora calospora				+	2.04%	8.77%	5.41%
Scutellospora dipapillosa			+		3.27%	9.09%	6.18%
Scutellospora persica	+				0.82%	2.11%	1.46%
Acaulospora bireticulata	+				6.53%	16.84%	11.69%
Acaulospora excavata				+	1.22%	5.26%	3.24%
Acaulospora rehmii	+				1.63%	4.21%	2.92%
Acaulospora rugosa		+			1.02%	5.00%	3.01%
Diversispora spurcum		+			2.04%	10.00%	6.02%
Paraglomus brasilianum			+		1.63%	4.55%	3.09%
Ambispora jimgerdemannii			+		6.94%	19.32%	13.13%

Note: AMF, arbuscular mycorrhizal fungi; DL, Dulan; GY, Guyuan; IF, isolation frequency, IV, importance value; JH, Jinghe; RA, relative abundance; ZN, Zhongning.

Table S3 Classification of AMF species for morphological identification 2

Species	Regions				IF	RA	IV
	ZN	GY	JH	DL			
SP1	+				1.63%	4.21%	2.92%
SP2			+		0.82%	2.27%	1.54%
SP3			+		2.45%	6.82%	4.63%
SP4				+	0.82%	3.51%	2.16%
SP5	+		+		2.45%	3.28%	2.86%
SP6		+	+		2.65%	4.71%	3.68%
SP7			+		0.82%	2.27%	1.54%
SP8		+	+		1.84%	3.26%	2.55%
SP9	+				0.82%	2.11%	1.46%
SP10	+				1.22%	3.16%	2.19%
SP11	+			+	2.04%	3.29%	2.67%
SP12		+			2.04%	8.77%	5.41%
SP13	+				0.82%	3.51%	2.16%
SP14	+				0.41%	1.75%	1.08%
SP15	+				1.22%	5.26%	3.24%
SP16	+				0.41%	1.75%	1.08%
SP17		+			2.04%	10.00%	6.02%
SP18				+	3.27%	14.04%	8.65%
SP19				+	1.22%	5.26%	3.24%
SP20			+		0.82%	2.27%	1.54%
SP21	+				1.22%	5.26%	3.24%
SP22	+				1.22%	5.26%	3.24%
SP23				+	0.82%	3.51%	2.16%
SP24		+			1.02%	5.00%	3.01%
SP25		+			1.02%	5.00%	3.01%
SP26		+			2.04%	10.00%	6.02%

Note: AMF, arbuscular mycorrhizal fungi; DL, Dulan; GY, Guyuan; IF, isolation frequency, IV, importance value; JH, Jinghe; RA, relative abundance; ZN, Zhongning.

Table S4 AMF species categorized by reference to morphology I-III, Species-specific labeling.

Number	Species	Number	Species	Number	Species
I-A	*Glomus aureum*	I-S	*Scutellospora dipapillosa*	II-Q	*SP10*
I-B	*Glomus badium*	I-T	*Scutellospora persica*	II-R	*SP11*
I-C	*Glomus coronatum*	II-A	*Acaulospora bireticulata*	II-S	*SP12*
I-D	*Glomus deserticola*	II-B	*Acaulospora excavata*	II-T	*SP13*
I-E	*Glomus etunicatum*	II-C	*Acaulospora rehmii*	III-A	*SP14*
I-F	*Glomus globiferum*	II-D	*Acaulospora rugosa*	III-B	*SP15*
I-G	*Glomus intraradices*	II-E	*Diversispora spurcum*	III-C	*SP16*
I-H	*Glomus melanosporum*	II-F	*Paraglomus brasilianum*	III-D	*SP17*
I-I	*Glomus microcarpum*	II-G	*Ambispora jimgerdemannii*	III-E	*SP18*
I-J	*Glomus minutum*	II-H	*SP1*	III-F	*SP19*
I-K	*Glomus monosporum*	II-I	*SP2*	III-G	*SP20*
I-L	*Glomus mosseae*	II-J	*SP3*	III-H	*SP21*
I-M	*Glomus pansihalos*	II-K	*SP4*	III-I	*SP22*
I-N	*Glomus pustulatum*	II-L	*SP5*	III-J	*SP23*
I-O	*Glomus reticulatum*	II-M	*SP6*	III-K	*SP24*
I-P	*Rhizophagus aggregatus*	II-N	*SP7*	III-L	*SP25*
I-Q	*Septoglomus constrictum*	II-O	*SP8*	III-M	*SP26*
I-R	*Scutellospora calospora*	II-P	*SP9*	III-N、O	AMF spores

Note: AMF, arbuscular mycorrhizal fungi.

Table S5 Effect of inoculation on element content of *Lycium barbarum*

Elemental content (mg/g)	K	Mg	Na
CK	51.096±3.588 b	13.928±1.875 a	73.232±10.272 a
G	63.333±3.899 ab	17.031±2.142 a	72.015±8.120 a
P	66.016±2.650 a	14.371±0.423 a	63.515±6.713 a
G×P	56.172±5.933 ab	16.139±1.375 a	69.915±4.673 a

Note: The inoculated *Glomus* Chen14 DL-Glo31 is abbreviated as *Glo*, *Paraglomus* sp. VTX00375 is abbreviated as *Par*, and the mixture of the two is *Glo×Par*, CK is the control. P<0.05. A, B and C represent the determination of the root ecological parameters index.

Table S6 Effect of inoculation on fresh weight of *Lycium barbarum*

Fresh weight (g/plant)	RFW	SFW	TFW
CK	0.063±0.011 b	1.712±0.193 a	1.774±0.203 a
G	0.040±0.012 b	1.543±0.262 a	1.582±0.272 a
P	0.129±0.017 a	2.013±0.226 a	2.142±0.211 a
G×P	0.066±0.005 b	2.144±0.392 a	2.210±0.387 a

Note: RFW, root fresh weight; SFW, shoot fresh weight; TFW, total fresh weight. The inoculated *Glomus* Chen14 DL-Glo31 is abbreviated as *Glo*, *Paraglomus* sp. VTX00375 is abbreviated as *Par*, and the mixture of the two is *Glo×Par*, CK is the control. P<0.05. A, B and C represent the determination of the root ecological parameters index.

Table S7 Effect of inoculation on dry weight of *Lycium barbarum*

Dry weight (g/plant)	RDW	SDW	TDW
CK	0.015±0.005 b	0.178±0.013 a	0.192±0.017 b
G	0.025±0.002 ab	0.178±0.024 a	0.202±0.022 ab
P	0.030±0.006 a	0.245±0.026 a	0.274±0.024 a
G×P	0.019±0.002 b	0.214±0.031 a	0.233±0.029 b

Note: RDW, root dry weight; SDW, shoot dry weight; TDW, total dry weight. The inoculated *Glomus* Chen14 DL-Glo31 is abbreviated as *Glo*, *Paraglomus* sp. VTX00375 is abbreviated as *Par*, and the mixture of the two is *Glo×Par*, CK is the control. P<0.05. A, B and C represent the determination of the root ecological parameters index.

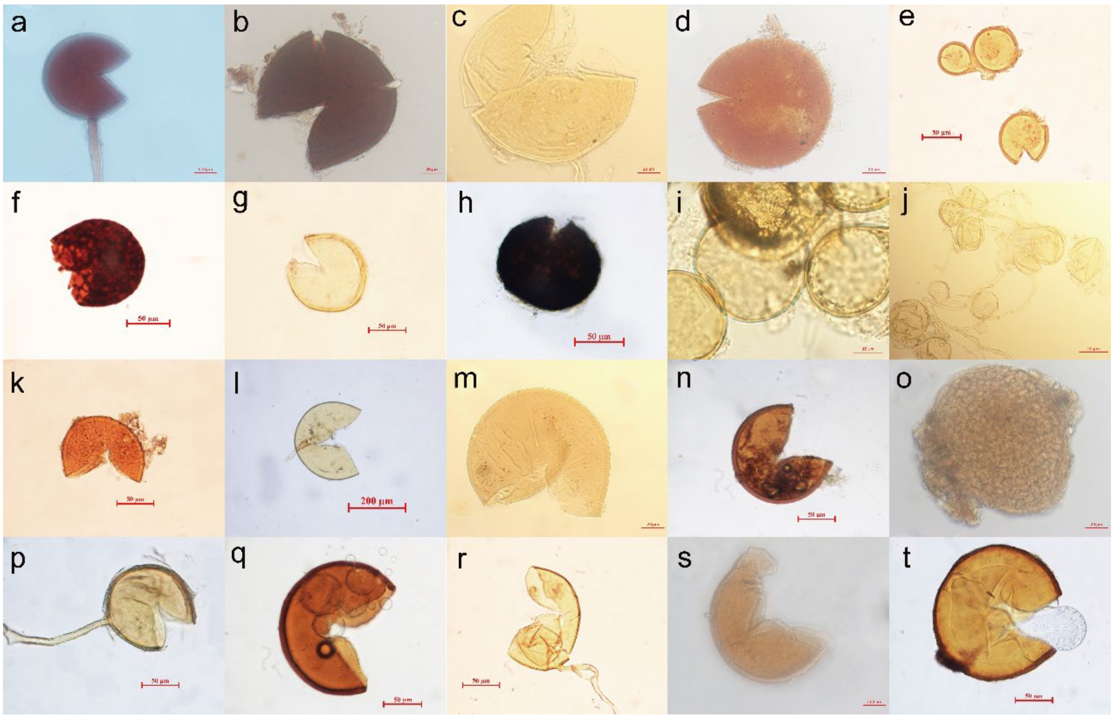

Figure S1. AMF species categorized by reference to morphology I. Detailed AMF species results query is shown in Table 1.

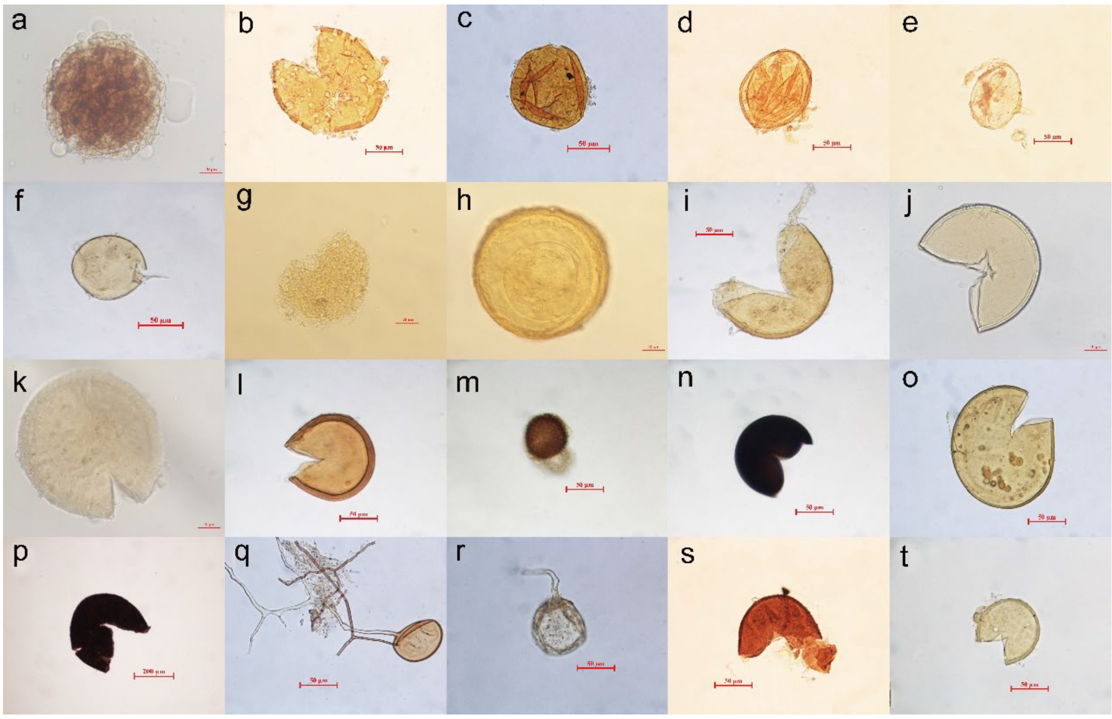

Figure S2. AMF species are categorized by reference to morphology II. Detailed AMF species results query attached Table 1. AMF, arbuscular mycorrhizal fungi.

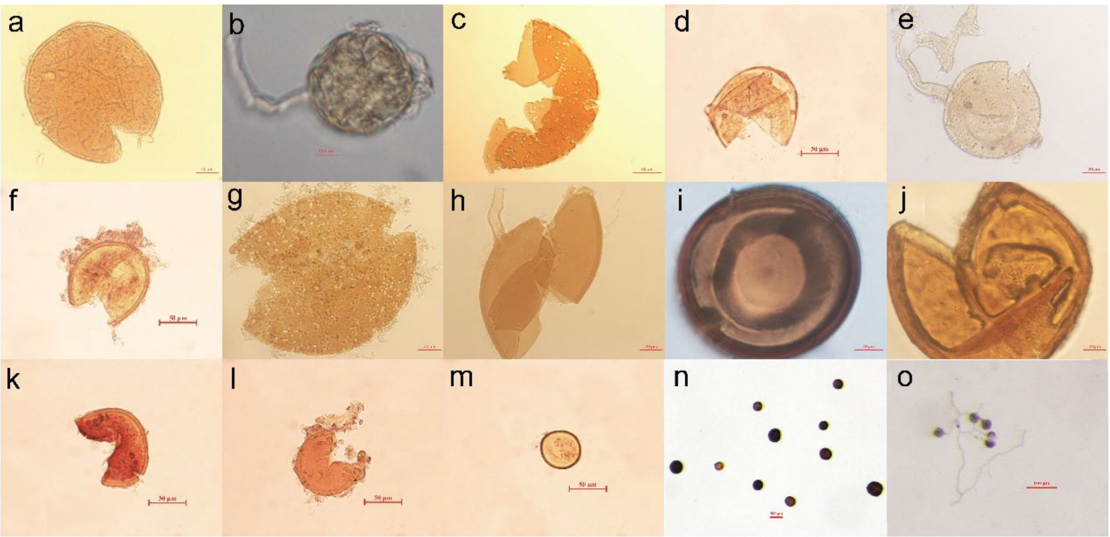

Figure S3. AMF species categorized by reference to morphology III. Detailed AMF species results query attached in Table 1., N and O is AMF spores under a 10x magnification field of view. AMF, arbuscular mycorrhizal fungi.
